# Two Cheers for the Cognitive Irregulars: Intelligence’s Contributions to Ageing Well and Staying Alive

**DOI:** 10.3390/jintelligence9030041

**Published:** 2021-08-18

**Authors:** Ian J. Deary

**Affiliations:** Department of Psychology, University of Edinburgh, Edinburgh EH8 9JZ, UK; i.deary@ed.ac.uk

**Keywords:** intelligence, mental tests, cognitive ageing, cognitive epidemiology, mortality

## Abstract

Here, intelligence is taken to mean scores from psychometric tests of cognitive functions. This essay describes how cognitive tests offer assessments of brain functioning—an otherwise difficult-to-assess organ—that have proved enduringly useful in the field of health and medicine. The two “consequential world problems” (the phrase used by the inviters of this essay) addressed in this article are (i) the ageing of modern societies (and the resulting increase in the numbers of people with ageing-related cognitive decrements and dementias) and (ii) health inequalities, including mortality. Cognitive tests have an ubiquitous place in both of these topics, i.e., the important fields of cognitive ageing and cognitive epidemiology, respectively. The cognitive tests that have sprouted in these fields are often brief and not mainstream, large psychometric test batteries; I refer to them as ‘irregulars’. These two problems are not separate, because results found with mental/cognitive/intelligence tests have produced a growing understanding that intelligence and health have a reciprocal, life-long relationship. Intelligence tests contribute to the applied research that is trying to help people to stay sharp, stay healthy, and stay alive.

I applaud the organisers of this symposium for asking us to address world problems to which intelligence can contribute and also, “to keep this symposium totally nonideological with respect to theories of, and methods for studying intelligence… to take whatever theory one has, or really, no theory at all… The symposium also would be nonideological with regard to political issues…” Good—let us do that. The request gave me the relief that Winston Churchill (1898) stated when he wrote, “I pass with relief from the tossing sea of Cause and Theory to the firm ground of Result and Fact.” I shall eschew the use of theory, as I explain elsewhere (Deary and Sternberg 2021); here, ‘intelligence’ will be represented by the scores obtained from cognitive tests *qua* readouts of brain functioning and predictors of future medical states.

Having applauded the organisers, I shall also apologise to Robert Sternberg—one of the organisers—because I turned down the invitation to write this piece a few times before accepting it. There did not seem to be much more for me to say by way of synoptic writing on intelligence; I had recently reviewed our work on the Lothian Birth Cohorts’ contributions to cognitive ageing (Corley et al. 2018), I had reviewed work on the genetic and neuroimaging associations with intelligence (Deary et al. forthcoming), I had reviewed the field of cognitive epidemiology (Deary et al. 2021), and I had massively revised/updated my wee, lay book on intelligence (Deary 2020). I had even had an extended—and good-natured—argument about aspects of intelligence with Robert Sternberg (Deary and Sternberg 2021). What else did I have to say? What eventually occurred to me was something that was closer to my medical origins—I used to be a medical doctor—rather than my adopted profession of psychology. It was that, by comparison with the sometimes-fraught topic of intelligence/cognitive testing in psychology and social sciences (why else would Warne (2020) have had to enumerate, explicate, and perhaps explode 35 myths about intelligence?), cognitive testing in medical/ageing/life-course research settings is almost omnipresent, stands usefully in line alongside other bodily assessments (Lara et al. 2015), is often done by rinky-dink-looking tests, and appears to make a modest but useful contribution to the understanding of some important human problems.

Here are the problems: why do some people live longer than others? Of those who survive to a given older age, why do some people’s cognitive functions, brains, and bodies age better than others? Longer lives are not necessarily better; the issue is that there are inequalities/differences/unfairnesses in people’s longevity. Older people are not a problem; the issue is that some things happen more to older people—illness, cognitive decline, loneliness, etc.—and there are inequalities in these happenings. These issues sit alongside the facts of there being greater proportions of older people, especially in so-called developed/higher-income nations, and that people are living longer nowadays.

Note that intelligence (psychometric cognitive test scores; see Deary 2020; Deary and Sternberg 2021) has a role as both exposure/independent and outcome/dependent variables. In this piece, I want to say only three things with respect to the contribution of intelligence to the issues of cognitive ageing and cognitive epidemiology. First, the less big-headed (pun intended) intelligence appears with respect to its contribution in addressing a problem, the better I like it; that is, no human problem is likely to be explicable by a single variable, nor described by a single variable. Intelligence makes modest contributions to studying some human problems. It is good and correct, I think, when intelligence—either as an exposure or an outcome—is a modest part of a collective or recipe. Intelligence makes contributions alongside other variables. Intelligence is never the only relevant thing, nor highly predictive on its own for an individual. Second, although we cognitive scientists would advocate using well-validated and detailed batteries of cognitive tests, it is surprising how useful short cognitive assessments have been and still are. Third, in medically-oriented cohort studies, cognitive testing is widespread and used, quietly and mostly happily, alongside other assessments of bodily functioning. Having said these three things briefly, I shall now say them at greater length.

## 1. A Modest Contribution

Disclaimer #1: Here, I want to take a wide-angled shot of intelligence and its place alongside other factors in the important assessment of health and ageing, a bit like a class photo of cognitive testing alongside its schoolmates in human assessment rather than a close-up portrait.

I wanted to begin by getting away from any chest thumping regarding intelligence. I found an example in a modest (for those who pay attention to impact factors) journal (Nilsen et al. forthcoming). It is a study of 674 Swedish people. It measured people’s job conditions five times between the ages of 25 years and 50, assessing the extent to which the participants were in intellectually demanding work or physically demanding, hazardous/stressful work. The authors found that, by the time people were over 70, those who started in more intellectually demanding work, and who accumulated more of it, tended to have more “successful ageing”. Successful ageing sounds important to me; therefore, let us look at how it was operationalised. The authors’ conglomerate measure included assessments of: social activities (meeting people in different situations), cultural activities, physical functioning, cognitive functioning, and absence of diseases. The importance is not so much in the results of the paper, more that this combination of being engaged mentally and socially, sharp physically, and medically well is often used to capture healthy ageing (e.g., Lara et al. 2015); they might have added happiness/psychological wellbeing, but we sha’n’t quibble.

The assessment of cognitive functioning was the abridged version of the Mini-Mental State Examination (Folstein et al. 1975; an already-short test, before the abridging), which involved immediate and delayed recall of three words; being oriented to year, month, date, country; and taking away 7 from 100 and repeating the subtraction another 5 times. That assessment of ‘intelligence’ would have taken just a few minutes; yet, there it was, as part of the assessment of the summum bonum of the human life course: healthy ageing. This small example made me think of two general issues with respect to intelligence/cognitive testing and its reciprocal association with health across the human life course: the modest cognitive assessments that are often made; and the widespread embedding of cognitive tests in life-course studies.

## 2. Three Pages of Cognitive Testing

Disclaimer #2: Yes, of course, one can apply one of the Rolls-Royce cognitive instruments (e.g., the full Wechsler or Stanford–Binet scales’ most up to date versions) to a person to obtain an assessment of their general, domain, and individual test levels of cognitive capability. Where I have been in charge of studies, I have tried to do that—using multiple tests for each of the cognitive domains—as far as was compatible with what time was available and what we thought participants would tolerate (Taylor et al. 2018). Where time has been more restricted, I have designed shorter batteries with single tests assessing important cognitive domains (Smith et al. 2012). However, oddly, the contribution of cognitive tests to the world issues of unequal longevity and unequal cognitive ageing has often been with briefer assessments. Here are three examples.

*The Binet–Simon Test*: An important time at which to assess cognitive functions, it was decided, when intelligence tests were invented, was in childhood; they might be used as a guide to the likelihood of an individual benefitting from a standard or non-standard educational environment. That is, some children might need more help to keep up, or some might need more stimulation if they are running ahead. As every schoolchild knows, the inventor of intelligence tests was Alfred Binet and, spread across pages 238–239 of his *Development of Intelligence* (Binet and Simon 1916)—but taking up just about one page in total (of course, the tester’s instructions run to far more than that)—are the 58 questions that assess the intelligence level of every age of child from 3 to 13 years. It might not have come to much, had Dr Ovide Decroly not handed Dr Henry Herbert Goddard the sheet of paper on which the test was printed (Zenderland 1998). The Stanford revision of the Binet–Simon test was used to validate Professor Sir Godfrey Thomson’s Moray House Test No. 12 (MHT) (Scottish Council for Research in Education 1933); the MHT and Binet tests correlated about 0.8. The MHT correlates (at a bit less than 0.2) with how long children live; a child with a 15-point (one standard deviation) advantage MHT score at age 11 years is, on average, about a quarter less likely to have died by their late 70s (Calvin et al. 2017). If we split the MHT scores from age 11 years into deciles, the lowest scoring decile is almost three times more likely to have died by their late 70s. Intelligence test score is neither a large nor the only contributor to survival across almost seven decades (Deary et al. 2021); however, it is remarkable that a 45-min paper and pencil test (the test is printed over five pages) of thinking skills has this predictive power for longevity.

*The MoCA (Monteral Cognitive Assessment; other cognitive assessments in older age are available; quite a lot of them, actually)*: It is important to assess cognitive functions in older age. Low cognitive function—especially if it is declining beyond a certain trajectory—can herald dementia or so-called ‘mild’ cognitive impairment (Tucker-Drob 2019). Outside of cognitive pathology, lowered cognitive functions are predictors of healthy older people dealing less well with the tasks of living and being independent (Tucker-Drob 2011). Losing cognitive capability is a feared aspect of growing old. In this important setting—another contribution of intelligence to world problems—one, again, sees wide use of tests that appear on a single page (of A4 paper, or American letter size). Google ‘moca test’ and click images and there it is, on one page: the MoCA test, including assessments of visuospatial-executive function, naming, memory, attention, language, abstraction, delayed recall, and orientation (Nasreddine et al. 2005). It has high sensitivity and specificity in detecting cognitive impairment. It correlated 0.64 with the WAIS Full Scale Intelligence Quotient (Sugarman and Axelrod 2014). My point is not that such short scales are perfect—far, far from it; rather, it is that these remarkably short scales have a place in assessing a large clinical issue and are very widely applied. The phenomenal success of the even-shorter Mini-Mental State Examination (Folstein et al. 1975) could also be mentioned; again, you can find that as a single-page image. The MMSE correlated 0.51 with a WAIS Full Scale Intelligence Quotient (Sugarman and Axelrod 2014).

*The NART (National Adult Reading Test)*: We have seen that one-page (more or less) tests can usefully help to conduct the important tasks of assessing cognitive function in childhood—related to educational needs and longevity (cognitive epidemiology)—and in older age—related to possible cognitive pathology (cognitive ageing) and managing one’s daily affairs. Wouldn’t it be handy, though, if, in older age, and even in the early stages of dementia, it was possible to estimate what people’s cognitive capabilities used to be? We know that crystallised capabilities, such as vocabulary, do not show the same decline in mean age trends as do more fluid capabilities, such as processing speed, reasoning, and aspects of memory (e.g., Salthouse 2009). However, Hazel Nelson (1982); Nelson and Willison (1991) took this a stage further. She devised a test—with stimuli printed on one page—that estimated prior peak cognitive ability; it is called the National Adult Reading Test (NART). It is based on a person’s ability to pronounce fifty words that do not follow the usual rules of English language grapheme–phoneme correspondence and/or stress. It works; that is, the score on this one-page test, which takes a couple of minutes to administer, can retrodict, pretty well, prior intelligence in healthy older people. The NART, administered at age 77 years, correlated 0.72 with the Terman-Merrill revision of the Binet Test that had been administered at age 11 years (Deary and Brett 2015); yes, 68 years previously. The MHT, administered at age 11 years, correlated 0.68 with the NART administered at age 70 years, and 0.66 with the Wechsler Test of Adult Reading (the forerunner of the Wechsler (2011) Test [sic] of Prefrontal Function) (Dykiert and Deary 2013). The correlation between NART (tested at age 79 years) and MHT (tested at age 11 years) was very similar—0.63 and 0.60, respectively—in people with and without dementia (McGurn et al. 2004). It is a testament to the usefulness of the idea of premorbid/prior intelligence estimation underpinning the NART that a cognitive testing juggernaut—Wechsler–Pearson–PsychCorp—then constructed its own test based on the same idea, i.e., the Test Of Premorbid Function; a quibble is that the word ‘Test’ would possibly be better renamed as ‘Estimator’ (Wechsler 2011).

## 3. Fifty Million Frenchmen Can’t Be Wrong

Disclaimer #3: It is optimal, probably, to do a fuller, more detailed test battery of cognitive functions; however, many, many studies from around world are showing that doing something by way of even the briefest cognitive testing is probably better than nothing. This is not an advocacy for not doing cognitive testing as well as possible; what it is is an appreciation of how, when one looks at human life-course studies, cognitive testing is almost everywhere and is trying to do a decent job of assessing something important about human brain functioning. Here, I give three examples that exemplify the cognitive footsoldiers/irregulars that occur in many studies around the world.

*UK Biobank*: This large population sample—admittedly non-representative of the United Kingdom’s middle-aged and older population (Frey et al. 2017)—has rightly become famous for its originators’ foresight in realising how informative and productive for human health half a million subjects—aged 40–70 years at recruitment and with medical, psycho-social, physiological, and genetic information—would be; oh, and with cognitive data, too. Almost all participants allowed their medical data to be collected until their death. To begin with, at their initial, approximately 90-min in-person assessment, the participants (I am one of them) took a go/no-go reaction time test and a memory test which, together, lasted just a couple of minutes. The reaction time had only four trials that counted for analysis. That makes it one more trial than in Clark Wissler’s (1901) study that supposedly ended James McKeen Cattell’s interest in cognitive testing; I say supposedly, because those who wrote about the study made conclusions that were not supported by its data (Deary 1994). Other overlaps between the UK Biobank’s baseline assessment and Cattell’s (1890) suggestion that cognitive tests would be useful if tested on large samples are the inclusion of hand-grip strength (dynamometer pressure) and immediate memory in the UK Biobank’s assessments. The UK Biobank’s memory test had to do with trying to remember where, in a matrix, there were pairs of pictures of objects; there were two trials: a three by two matrix with three pairs and a four by three matrix with six pairs. For the last third of the baseline testing of the original 500,000 participants in the UK Biobank, a test called ‘Fluid’ was introduced. It was a test of verbal and numerical reasoning (VNR), with 13 items, and a maximum time of two minutes for completion. A few more tests were popped into the small battery at that time, and I and a colleague introduced some more later (Fawns-Ritchie and Deary 2020). I tend to veer toward more extensive cognitive batteries with longer and more diverse tests when I advise on cognitive testing in population cohorts; however, it was interesting to see how some of the UK Biobank’s tests—including the two-minute fluid/VNR test—correlated with better-validated tests (Fawns-Ritchie and Deary 2020). What’s my point? The UK Biobank’s cognitive assessment was, originally, about as small a battery of cognitive tests as could be imagined; nevertheless, there have been many important publications based on this sample’s sparse cognitive data, including: health/dementia-related studies (e.g., Calvin et al. 2019); brain structure–cognition correlation studies in older age (Cox et al. 2019); and the largest genome-wide association studies of intelligence (Hill et al. 2019; Davies et al. 2018; Savage et al. 2018; UK Biobank’s genetic and small-scale cognitive data were the mainstay of all of these three, being a large majority of the human samples in each of these studies). Don’t take my word for it based on the few papers I just cited; just type in ‘UK Biobank cognitive’ to PubMed and see how many there are, or go to the UK Biobank’s publications page and type in ‘cognitive’ (https://www.ukbiobank.ac.uk/enable-your-research/publications), and you will find many publications including, for example, the association between baseline cognitive ability and COVID-19 mortality (Batty et al. 2021); there has been a huge payback for a few minutes’ worth of cognitive testing.

*The HRS Family of studies*: The CANDID initiative (De Looze et al. 2021)—‘Leveraging Cognitive Ageing Dementia Data from around the World’—was conducted by researchers from The Irish Longitudinal Study on Ageing (TILDA; Donoghue et al. 2018). It is an example of how community-based population cohorts of older people tend to include cognitive data as an important part of their broad, health-related phenotyping. The CANDID report included samples from China (N = 17,500+), Costa Rica (7000+), England (18,000+), Brazil (~10,000), the USA (30,000+), Japan (~4200), Korea (10,000+), India (~50,000), Mexico (15,000+), Northern Ireland (8500), Europe (27 countries contributing 140,000+), and the Republic of Ireland (8504). All included cognitive tests. The tests varied a lot, although most studies included a test of verbal declarative memory using immediate and delayed recall of items. There were, across all these studies, cognitive ability assessments including tests of global cognitive function (e.g., MMSE and MoCA), memory (five different types of tests), attention/working memory/executive function (a mixed bag, with tests including letter cancellation, visual scanning, digit span, trail making, choice reaction time, verbal fluency), numerical ability, and language skills. The long CANDID report presents the method of conducting each test and the comparability of assessments across studies; that is, for example, whether two memory tests could be considered to be assessing the same function. This moot issue was something that we tested empirically in the UK Biobank cognitive test validation sample (Fawns-Ritchie and Deary 2020).

*The CHARGE consortium, etc.*: This is just one example of the use of cognitive test data to examine the contribution of genetic variation to cognitive functions in mostly older people, which is a contribution to finding out what causes variation in cognitive functions in older age. In one study (Davies et al. 2018), which combined the UK Biobank with population-based cohorts from the CHARGE and COGENT consortia there were over 50 different population samples, all with cognitive testing, including samples from France, the United Kingdom (9 cohorts), Germany (4), Iceland, the USA (19), Austria (2), Greece (2), Australia (4), Sweden (4), Canada (2), Croatia (2), Republic of Ireland (2), The Netherlands (4), Finland (3), Denmark, and Norway (2). These included UK Biobank, but there was, otherwise, limited overlap with the HRS family of studies described above. These studies were included because they had at least three cognitive tests assessing different domains of cognitive function. This meant that, for each sample, principal components analysis could be applied to examine for a general cognitive function component that was used as the ‘common’ phenotype across the samples, based on the finding that general cognitive components derived from different batteries tend to correlate highly (Johnson et al. 2004).

Recognising that cognitive and other bodily markers were commonly used in life course and aging studies, a group of us put together some guidelines for which ones to choose (Mathers et al. n.d.; see Lara et al. 2015 for a shorter version). The suggested biomarkers included assessments of physical, cognitive, immune, endocrine, and physiological functions. We recognised the heterogeneity of the test batteries used across many dozens of international studies, and suggested possibilities for harmonising them; do have a look at Appendix 3 of the Mathers et al. report, which lists 16 “problems and considerations in establishing cognition-related biomarkers of healthy ageing” (yes, there will probably be more than that). The CANDID report (De Looze et al. 2021) was also an attempt at harmonising cognitive batteries. A related attempt to have standard measures out there for life course studies is the NIH Toolbox, which provides a set of health-related measures including cognitive and other bodily assessments, many of which apply from age 3 to 85 years; they are brief and have undergone rigorous psychometric assessment (Gershon et al. 2013, and see the NIH Toolbox brochure for cognition, emotion, sensation, and motor measures, and do compare it with Cattell (1890): https://www.healthmeasures.net/images/nihtoolbox/NIH_Toolbox_brochure_June_2017.pdf (accessed on 1 July 2021)).

## 4. Conclusions

The attempt here has been to show that intelligence—in the form of the scores from cognitive assessments—has a place in looking at the differences/inequalities in people’s cognitive ageing and in health differences/inequalities. It is not an argument for using quick-and-dirty cognitive assessments, although it is an appreciation of those who have usefully done so; I applaud the cognitive irregulars/contemptibles and their achievements. (One could make a similar argument—concerning the ubiquity of short measures in life-course cohort studies, alongside short cognitive tests—in other domains of testing. An exercise physiologist/physician might object that grip strength is too crude a measure of fitness, and a respiratory physiologist/physician might object that forced expiratory volume in one second [FEV1] is too crude a measure of lung function; however, both grip strength (Bohanon 2019) and FEV1 (Young et al. 2007) are used a great deal in cohort studies and are found to be useful (Lara et al. 2015).) In the spirit of ‘don’t make the perfect the enemy of the good’, it was demonstrated how even short cognitive tests—far from the detailed batteries we psychologists would prefer to have been used—appear regularly in medical settings to do with epidemiology and geriatric medicine. Theirs is a relatively quiet presence, and they appear in medical research that perhaps does not have the heat that intelligence and intelligence assessment can generate in the more purely psychological literature. The contributions of cognitive tests are modest in these life-course/medical/ageing settings: they are neither the only exposure of interest, nor the only outcome that matters. However, as the world deals with the change in age-demographic composition, and as health inequalities are increasingly recognised, cognitive testing has been woven into the fabric of human assessments that matter. Their omnipresence, almost, in population studies evidences their usefulness. Their quiet, effective, widespread contribution to studies of health and the human life course is like that of the United Kingdom’s Royal Regiment of Artillery (RA); the RA is no longer awarded battle honours and, instead, has the motto ‘Ubique’: everywhere.

## Data Availability

Not applicable.
